# Coronavirus membrane-associated papain-like proteases induce autophagy through interacting with Beclin1 to negatively regulate antiviral innate immunity

**DOI:** 10.1007/s13238-014-0104-6

**Published:** 2014-10-15

**Authors:** Xiaojuan Chen, Kai Wang, Yaling Xing, Jian Tu, Xingxing Yang, Qian Zhao, Kui Li, Zhongbin Chen

**Affiliations:** 1 Division of Infection and Immunity, Department of Electromagnetic and Laser Biology, Beijing Institute of Radiation Medicine, 100850 Beijing, China; 2 Department of Immunology, Bengbu Medical College, 233030 Bengbu, China; 3 Prince of Wales Clinical School, The University of New South Wales, 2052 Sydney, NSW, Australia; 4 Department of Microbiology, Immunology and Biochemistry, University of Tennessee Health Science Center, 38163 Memphis, TN, USA

**Keywords:** coronavirus, papain-like protease, autophagy, antiviral immunity, Beclin1, STING

## Abstract

Autophagy plays important roles in modulating viral replication and antiviral immune response. Coronavirus infection is associated with the autophagic process, however, little is known about the mechanisms of autophagy induction and its contribution to coronavirus regulation of host innate responses. Here, we show that the membrane-associated papain-like protease PLP2 (PLP2-TM) of coronaviruses acts as a novel autophagy-inducing protein. Intriguingly, PLP2-TM induces incomplete autophagy process by increasing the accumulation of autophagosomes but blocking the fusion of autophagosomes with lysosomes. Furthermore, PLP2-TM interacts with the key autophagy regulators, LC3 and Beclin1, and promotes Beclin1 interaction with STING, the key regulator for antiviral IFN signaling. Finally, knockdown of Beclin1 partially reverses PLP2-TM’s inhibitory effect on innate immunity which resulting in decreased coronavirus replication. These results suggested that coronavirus papain-like protease induces incomplete autophagy by interacting with Beclin1, which in turn modulates coronavirus replication and antiviral innate immunity.

## Introduction

Autophagy is an evolutionarily conserved intracellular process which plays an important role in eliminating damaged organelles and long-live proteins for maintenance of cellular homestasis (Mizushima, 2009). During autophagy, cytoplasmic constituents are engulfed by double-membrane vesicles termed autophagosomes that are destined for fusion with lysosomes for content degradation. Under stress conditions such as starvation, autophagic acitivity is greatly augmented from its basal levels, providing recyclable resources that help maintain metabolism and ATP levels for cell survival (Rabinowitz and White, 2010). Recent studies have demonstrated that autophagy is involved in various physiologic and pathologic processes including cancer, cardiovascular disease, metabolism, viral infections and immune response, etc. (Deretic, 2012; Levine and Deretic, 2007; Mihaylova and Shaw, 2011; Shintani and Klionsky, 2004; Yang et al., 2011).

Autophagy is a dynamic process which includes initiation, nucleation, elongation and maturation. It is tightly regulated by a number of autophagy-related genes (*ATG*) and other multiple protein complexes (Kroemer and Levine, 2008). The ULK1-Atg13-FIP 2000 and class III PI3K complex, which contain Beclin1 and vps34, among other key components, regulate the initiation and nucleation of phagophore, the precursor of autophagic membranes. In the stage of elongation and complete closure of autophagosome membranes, two ubiquitin-like conjugation systems come into play. The first one is the Atg12-Atg5-Atg16L complex conjugation system and the second involves Atg4B, Atg3 and Atg7. The net outcome of the ubiquitin-like reactions catalyzed by these two systems is the conversion of microtubule associated protein 1B light chain 3 (LC3) from its cytosolic form (LC3-I) to the membrane bound, phosphatidylethanolamine conjugated form (LC3-II), a process known as LC3 lipidation. The conversion of LC3-I to LC3-II is a hallmark of autophagy and is indicative of autophagic activity, which can be monitored by LC3 immunoblotting or immunofluorescence (to monitor LC3 puncta formation) (Barth et al., 2010; Choi et al., 2013; Kabeya et al., 2000; Mizushima et al., 2010). The terminal step of autophagy, in which autophagosome is fused with lysosomes for content degradation, is assisted by UVRAG, VPS34, VPS15 and Beclin1 complex (Glick et al., 2010; Kuballa et al., 2012; Weidberg et al., 2011).

During their co-evolution with hosts, viruses have acquired elaborate strategies to evade, counteract and sometimes co-opt protective mechanisms of host cells. Unfortunately, the autophagy pathway is one of these. While this lysosomal degradation pathway can lead to degradation of viral components, acting as an intrinsic antiviral defense mechanism, many viruses either block autophagy or exploit it for their own replication (Dong and Levine, 2013; Dreux and Chisari, 2010; Richards and Jackson, 2013; Sumpter and Levine, 2011). As a critical regulator in both the early and late steps of autophagy, Beclin1 has emerged as a prime target for different viruses, such as influenza virus, HIV, and herpes viruses (Munz, 2011). However, targeting Beclin1 by a positive-strand RNA virus has not been reported. Coronaviruses (CoVs) are a large group of positive-strand RNA viruses that replicate in cytoplasm of host cells but don’t induce high level of IFN products (Clementz et al., 2010; Devaraj et al., 2007; Zhou and Perlman, 2007). These viruses induce the formation of cytoplasmic double-membrane vesicles (DMVs) which are believed to be the site for assembly of viral transcription-replication complexes (Gosert et al., 2002; Hagemeijer et al., 2010; Knoops et al., 2008; Snijder et al., 2006). The origin of DMVs remains obscure, but these structures are often decorated with an autophage marker, LC3 (Bernasconi et al., 2012; Maier and Britton, 2012; Maier et al., 2013). Although coronavirus infections are known to be accompanied with activation of autophagy (Cottam et al., 2011; Knoops et al., 2008; Prentice et al., 2004; Reggiori et al., 2010), relatively little is known about the role autophagy may play in the coronavirus life cycle, as are the mechanisms of autophagy induction and its contribution(s) to viral regulation of host responses, especially those concerning innate immunity. Overall, these aspects have been under intense investigation, and new knowledge stemmed from such studies hold the key(s) to a better understanding of coronvirus pathogenesis.

Previously, it has been shown that the papain-like protease (PLP) domains contained in the nonstructural protein (nsp) 3 of coronaviruses of SARS-CoV (Devaraj et al., 2007), HCoV-NL63 (Clementz et al., 2010) and porcine epidemic diarrhea Virus (PEDV) (Xing et al., 2013) act as IFN antagonists. Several mechanisms have been proposed. First, the SARS-CoV PLpro forms a complex with IRF3 and blocks IRF3 phosphorylation and nuclear translocation (Devaraj et al., 2007). Second, SARS-CoV PLP and PLP2 of HCoV-NL63 and PEDV also interact with and impede the dimerization of stimulator of IFN genes (STING, a.k.a, MITA/MPYS/ERIS) (Sun et al., 2012a). Third, the aforementioned coronavirus PLPs possess de-ubiquitinating activity, which may also contribute to the ability of these PLPs to disrupt host innate immunity by removing ubiquitin moieties from signaling molecules in the innate antiviral pathways (Bibeau-Poirier and Servant, 2008; Chen, 2012; Loo and Gale, 2011). Given that autophagy is increasingly implicated in virus-host interactions and activation of this pathway is dependent on ubiquitin-like reactions, we set out to determine whether PLP impacts on autophagy. Our data show that the membrane-associated PLP2 (PLP2-TM) of HCoV-NL63 and PEDV and PLpro-TM of SARS-CoV and the new-emerging Middle East respiratory syndrome coronavirus (MERS-CoV) (Yang et al., 2014) represent a novel class of viral proteins encoded by coronaviruses that induce autophagy. Interestingly, we found that PLP2-TM induces incomplete autophagy, leading to accumulation of autophagosomes while impairing the fusion of autophagosomes with lysosomes. Intriguingly, PLP2-TM was found to associate with LC3 and Beclin1, two critical components of the autophagy pathway, and knockdown of Beclin1 partially reversed the blockade of PLP2-TM on innate immune signaling to the IFN-β promoter. The PLP-TM targeting of Beclin1 to control autophagic activity may represent a novel mechanism of coronaviral regulation of antiviral innate immunity.

## Results

### PLP2-TM is a novel autophagy-inducing protein encoded by coronavirus

**Figure 1:**
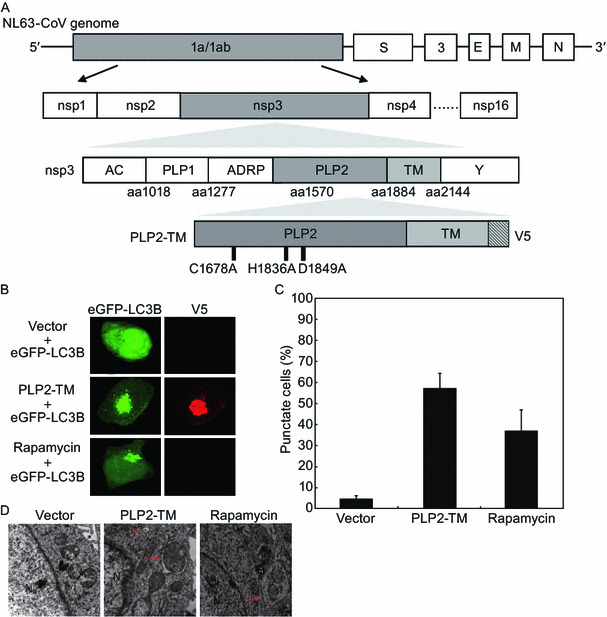
**PLP2-TM is a novel autophagy-inducing protein encoded by coronavirus**. (A) Schematic diagram illustrating coronavirus NL63 genome, polyprotein (pp) 1a/b, the predicted processing of replicase polyproteins (pp) to nsp’s. The domains, including the predicted transmembrane (TM), in nsp3, and the membrane anchored-PLP2 construct (PLP2-TM) that were used in this study are indicated. (B) PLP2-TM is a novel autophagy-inducing protein encoded by coronavirus. The plasmid of HCoV-NL63 PLP2-TM-V5 was co-transfected with pcDNA3.1-eGFP-LC3B into HEK2923T cells. As the positive control for induction of autophagy, HEK293T cells were transfected with eGFP-LC3B for 48 h and then treated with complete medium supplemented with 400 nmol/L Rapamycin for 6 h. The immunofluoresence of the cells were detected using a confocal microscope after stained with anti-V5-tagged primary antibody, followed by being stained with Alexa Fluor 594-conjugated goat anti-rabbit secondary antibody. The localization of eGFP-LC3B positive autophagosome accumulation (green) and the V5-tagged PLPs products (anti-V5, red) was visualized using a confocal microscope. (C) Quantification of cells displaying eGFP-LC3B puncta in PLP2-TM transfected cells. HEK 293T cells were transiently co-transfected with eGFP-LC3B and PLP2-TM-expression constructs. Forty-eight hours later, cells with eGFP-LC3B puncta formation were quantificated under a fluorescence confocal microscope. Three random fields, each containing at least 80 cells, were counted. Results from one representative experiment are shown in Fig. 1B. (D) PLP2-TM induces autophagosome-like structures detected using a transmission electron microscope. HEK293T cells were transfected with pcDNA3.1 or PLP2-TM-V5 for 48 h and then cells were analyzed for autophagosome formation using a transmission electron microscope. The cells treated by 400 nmol/L Rapamycin were analyzed to serve as a positive control for induction of autophagy. Red arrows indicate representative autophagosome-like structures. N indicates the cellular nuclear. Scale bar indicates 500 nm

To determine whether coronavirus PLPs regulate autophagy, we first studied the impact of ectopic expression of the transmembrane (TM)-containing form of PLP2 (PLP2-TM) of HCoV-NL63 on formation of autophagosomes (Chen et al., 2007; Clementz et al., 2010; Sun et al., 2012a) (Fig. 1A). HEK293T cells were co-transfected with an eGFP-LC3B-expressing plasmid and the V5 eiptope-tagged PLP2-TM. At 48 h post-transfection, cells were fixed, immunostained using an anti-V5 antibody, and examined for PLP expression and evidence of autophagosome formation by confocal fluorescence microscopy. As a positive control, cells transfected with eGFP-LC3B for 48 h were treated with Rapamycin, a well-known autophagy inducer, for 6 h. eGFP-LC3 fluorescence microscopy is a well-accepted method of monitoring autophagosome formation, as measured by a change in the eGFP-LC3 fluorescence pattern from diffuse to punctate distribution (Klionsky et al., 2012). As shown in Fig. 1B and Fig. 1C, HEK293T cells had very low basal autophagic activity, with less than 5% of cells exhibiting punctate eGFP-LC3. Short-term treatment with Rapamycin efficiently induced autophagy, resulting in eGFP-LC3 puncta formation in ~40% of cells. Ectopic expression of PLP2-TM caused a clear redistribution of eGFP-LC3B to punctate structures, indicative of formation of autophagosomes (Fig. 1B). Quantitative analysis revealed that while ~60% of cells expressing PLP2-TM exhibited eGFP-LC3 punctation (Fig. 1C). These results suggest that besides the deubiquitinase (DUB) activity and IFN antagonist (Barretto et al., 2005; Chen et al., 2007; Clementz et al., 2010; Devaraj et al., 2007; Sun et al., 2012a), the PLP2-TM encoded by human coronavirus NL63 is a novel autophagy-inducer.

To corroborate this finding, we performed transmission electron microscopy (TEM) to directly visualize autophagosomes in cells expressing PLP2-TM, and as a negative control, in cells expressing the empty vector. Double membrane vacuoles containing cytoplasmic organelles, which are characteristic of autophagosomes, were readily visible in the cytoplasm of PLP2-TM-transfected cells (Fig. 1D, middle panel), as were in cytoplasm of Rapamycin-treated cells (Fig. 1D, right panel). In contrast, no autophagosome-like structures were observed in cells transfected with the empty vector (Fig. 1D, left panel). The number of autophagic vacuoles per cell was significantly higher in the PLP2-TM-transfected cells as compared with that in the empty vector-transfected cells (Fig. 1D, middle panel vs. left panel). These data provide morphological evidence that PLP2-TM promotes autophagosome accumulation. In addition, they confirm that the autophagy-inducing effect was attributed to expression of PLP2-TM but not to the transfection reagent/process per se.

### Expression of PLP2-TM induces autophagy in multiple cell lines

**Figure 2:**
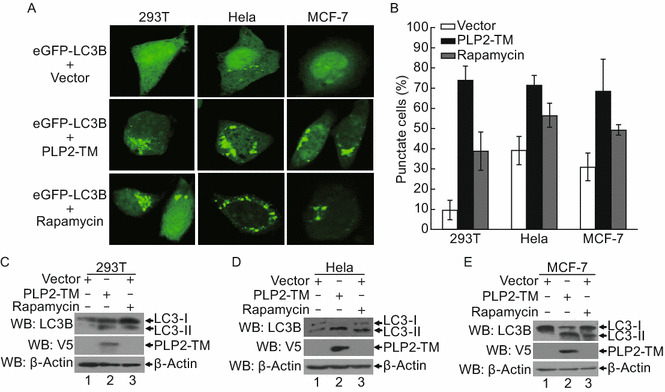
**Autophagosome induced by CoV NL63 PLP2-TM in various cell lines**. (A) PLP2-TM-V5 and pcDNA3.1-eGFP-LC3B were co-transfected respectively into HEK2923T, HeLa and MCF-7 cells. The cells were fixed at 48 h post-transfection and were analyzed for eGFP-LC3B positive autophagosome accumulation (green) using a confocal microscope as described in Fig. 1B. (B) Quantification of cells displaying eGFP-LC3B puncta from one representative experiment that shown in Fig. 2A as described in Fig. 1C. (C–E) HEK293T, HeLa and MCF-7 cells were transfectd with PLP2-TM-V5 or empty vector as a negative control for 48 h. These cells were also treated by 400 nmol/L Rapamycin to serve as a positive control for induction of autophagy. The cells were then lysed for Western blotting analysis using a rabbit anti-LC3 antibody to detect the endogenous LC3 expression (top panel in each Fig. 2C–E). The whole cell lysate (WCL) was blotted using anti-V5 antibodies to evaluate expression of PLP2-TM (middle panel in each Fig. 2C–E), and β-Actin was detected from whole cell lysate (WCL) as a loading control (bottom panel in each Fig. 2C to 2E)

Next, we investigated whether PLP2-TM induces autophagic vesicle formation in cell lines other than HEK293T cells. To this end, HeLa and MCF-7 cells were co-transfected with eGFP-LC3B and either PLP2-TM or the empty vector, followed by examination of eGFP-LC3 puncta formation. Both cell lines had higher basal levels of autophagic activity than HEK293T cells, as demonstrated by the presence of a small number of eGFP-LC3 puncta in 30%–40% of cells expressing the empty vector (Fig. 2A and 2B). The high basal levels of autophagic activity in control HeLa cells were not unexpected, as similar finding has been reported by others (Mizushima et al., 2010). Importantly, ectopic expression of PLP2-TM doubled the percentage of cells exhibiting eGFP-LC3 puncta in both HeLa and MCF-7 cells (Fig. 2B).

Activation of autophagy involves the association of LC3 with autophagic phagophore membranes that depends on the conversion from the cytosolic, soluble form, LC3-I, to the membrane-bound form, LC3-II. This ubiquitin-like modification, termed lipidation, can be illustrated by a change in migration on SDS-PAGE gel and is a widely used marker for monitoring autophagy (Klionsky et al., 2012; Mizushima et al., 2010). To further validate the finding that PLP2-TM promotes autophagy, we measured the expression levels of endogenous LC3-I and LC3-II in cells following expression of PLP2-TM, in comparison with cells expressing the control vector, by Western blotting. Consistent with the eGFP-LC3 fluorescence and TEM data, ectopic expression of PLP2-TM substantially increased the abundance of LC3-II in HEK293T (Fig. 2C), HeLa (Fig. 2D) and MCF-7 (Fig. 2E) cells. Collectively, these data confirm that the induction of autophagy by PLP2-TM is not a cell-type specific phenomenon. It is of interest to point out that the basal levels of endogenous LC3-II were higher in HeLa cells than in HEK293T cells, an observation which was in agreement with the data on eGFP-LC3 punctation (Fig. 2B).

### Induction of autophagy by various coronaviral PLPs in a protease-independent manner

**Figure 3:**
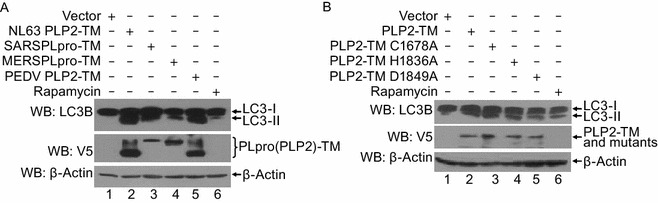
**Induction of autophagy by various coronaviral PLPs that is independent on the protease activity**. (A) The plasmids of HCoV-NL63 PLP2-TM, SARS-CoV PLpro-TM, MERS-CoV PLpro-TM and PEDV PLP2-TM were transfected into HEK293T cells. As the positive control for induction of autophagy, HEK293T cells were treated with complete medium supplemented with 400 nmol/L Rapamycin for 6 h. The cells were then lysed for Western blotting analysis using a rabbit anti-LC3 antibody to detect endogenous LC3 expression (top panel in Fig. 3A). The whole cell lysate (WCL) was blotted using anti-V5 antibodies to evaluate expression of PLP2 (PLpro)-TM (middle panel in Fig. 3A), and β-Actin was detected in whole cell lysate (WCL) as a loading control (bottom panel in Fig. 3A). (B) PLP2-TM-V5 and the catalytic mutants (C1678A, H1836A and D1849A) as showed in Fig. 1A were transfected respectively into HEK2923T, and induction of autophagy was detected as described in Fig. 3A

As with HCoV-NL63 PLP2-TM, the PLpro-TM of SARS-CoV, MERS-CoV PLpro-TM and PLP2-TM of PEDV also possess de-ubiquitinating and interferon antagonism activities (Clementz et al., 2010; Devaraj et al., 2007; Xing et al., 2013; Yang et al., 2014). The finding that PLP2-TM of HCoV-NL63 induces autophagy prompted us to investigate whether the PLP2-TM homologues encoded by other coronaviruses also have a similar function. Western blotting was carried out to determine the expression of LC3-I and LC3-II in HEK293T cells ectopically expressing various coronaviral PLPs (Fig. 3A). While LC3-II was undetectable in cells transfected with control vector (lane 1, Fig. 3), its expression was strongly induced in cells expressing SARS-CoV PLpro-TM (lane 3, Fig. 3A) or PEDV PLP2-TM (lane 5, Fig. 3A), as was in cells expressing HCoV-NL63 PLP2-TM (lane 2, Fig. 3). Although its effect was less pronounced, expression of the MERS-CoV PLpro-TM also caused substantial increase in LC3-II expression (lane 4, Fig. 3A). Next, to determine if the protease activity of PLP2 is required for its induction of autophagy, NL63 PLP2-TM and its catalytic mutants (C1678A, H1836A and D1849A) (Fig. 1A) were transfected into HEK293T cells and the conversion from the cytosolic, soluble form, LC3-I, to the membrane-bound form was detected. We found that, like as the PLP2-TM, ectopic expression of all the catalytic mutants substantially increased the abundance of LC3-II, indicating that the catalytic activity is not required for induction of autophagy by PLP2-TM. Collectively, these results imply that the ability to promote autophagy is a shared attribute of coronavirus PLP2-TM and its homologs in a protease-independent manner. In subsequent investigations, we focused our efforts on using HCoV-NL63 PLP2-TM to delineate the molecular details underlying interactions of coronaviral PLP with the autophagy pathway.

### Time-dependent induction of autophagy by PLP2-TM

**Figure 4:**
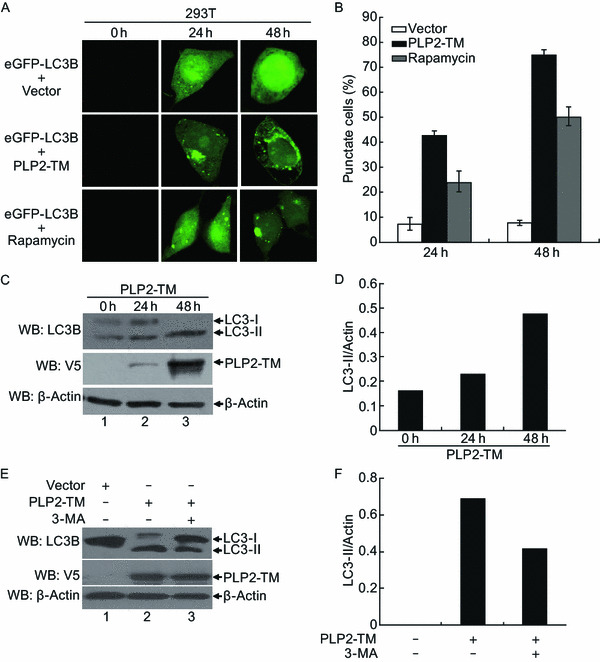
**PLP2-TM induces autophagy formation in a time-dependent manner**. (A) Immunofluorescence microscopy was used to detect the eGFP-LC3B-autophagosome in PLP2-TM transfected cells at different time point post-transfection. The plasmid of PLP2-TM-V5 was co-transfected with pcDNA3.1-eGFP-LC3B into HEK2923T cells. The cells were fixed at 0 h, 24 h and 48 h post-transfection, respectively and then analyzed for eGFP-LC3B positive autophagosome accumulation using a confocal microscope as described in Fig. 1B. (B) Quantification of cells displaying eGFP-LC3B puncta from one representative experiment that shown in Fig. 4A as described in Fig. 1B. (C and D) HEK293T was transfected with PLP2-TM-V5 or empty vector as a negative control. At 0 h, 24 h and 48 h post-transfection, the cells were then lysed for Western blotting analysis using a rabbit anti-LC3 antibody to detect the endogenous LC3 expression (top panel in Fig. 4C). The whole cell lysate (WCL) was blotted using anti-V5 antibodies to evaluate expression of PLP2-TM (middle panel in Fig. 4C), and β-Actin was detected in whole cell lysate (WCL) as a loading control (bottom panel in Fig. 4C). The band intensity was semi-quantitated by densitometric analysis using ImageJ software. (E and F) HEK293T cells were pretreated by 250 µmol/L 3-MA, an autophagy inhibitor, for 1.5 h and then transfected with PLP2-TM or empty vector for 48 h. The LC3, PLP2-TM and β-Actin were detected and semi-quantitated as described above

To understand the temporal regulation of autophagy by PLP2-TM, we monitored time-course eGFP-LC3B puncta formation in HEK293T cells co-transfected with HCoV-NL63 PLP2-TM and eGFP-LC3B. Significant eGFP-LC3B puncta formation was observed in cytoplasm of PLP2-TM-transfected cells as early as 24 h post transfection (Fig. 4A), when PLP2-TM protein was first detected (Fig. 4C). In addition, eGFP-LC3B puncta steadily increased at 48 h post transfection (Fig. 4A and 4B), concomitant with an increase in PLP2-TM expression levels (Fig. 4C). Approximately 40% of cells displayed cytoplasmic eGFP-LC3B puncta at 24 h postransfection, and that number climbed to >70% at 48 h (Fig. 4B). In contrast, cells co-transfected with control vector exhibited largely diffuse eGFP-LC3B fluorescence, with less than 7% of cells having background levels of eGFP-LC3B puncta, regardless of the time points monitored (at either 24 or 48 h post transfection, Fig. 4A and 4B). In line with these data, the abundance of endogenous LC3-II protein was reproducibly upregulated in a time-dependent manner in cells transfected with PLP2-TM (Fig. 4C and Fig. 4D), a phenomenon which was not observed with cells transfected with the control vector (data not shown). The increase in LC3-II protein levels by PLP2-TM, however, was substantially reduced in cells treated with 3-MA (Fig. 4E and 4F), an autophagy inhibitor (Mizushima et al., 2010), providing additional support for the notion that PLP2-TM increases cellular autophagic activity. In aggregate, the time-dependent increase in eGFP-LC3B puncta formation and upregulation of LC3-II abundance in PLP2-TM-transfected cells but not in control vector-expressing cells confirms that the heightened autophagic activity results specifically from PLP2-TM expression.

### PLP2-TM impairs the fusion of autophagosomes with lysosomes

**Figure 5:**
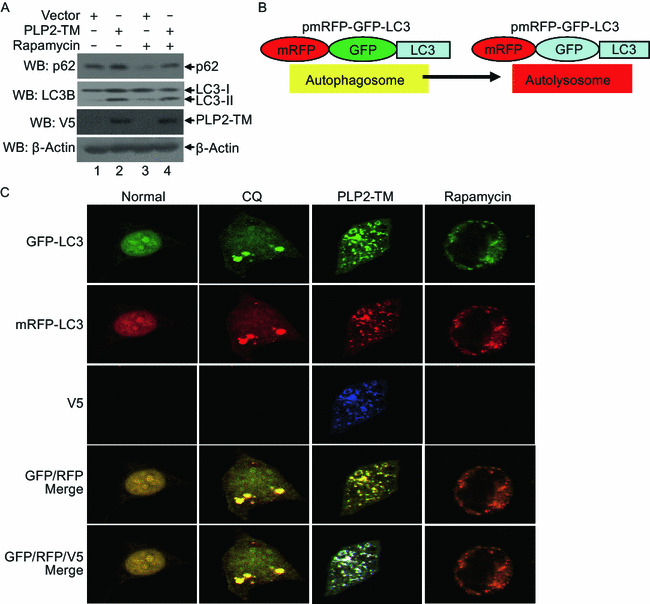
**PLP2-TM activates autophagosome formation but prevents its fusion with lysosomes**. (A) HEK293T cells were transfected with pcDNA3.1 or PLP2-TM. As the positive control for induction of autophagy, HEK293T cells were treated with complete medium supplemented with 400 nmol/L Rapamycin for 6 h. At 48 h post-transfection, the levels of endogenous autophagic substrate p62, LC3 protein and PLP2-TM were determined using Western blot analysis. Beta-actin expression was examined as a protein loading control. (B) Diagram of mRFP-GFP-LC3 structure and the principle for probing autophagy flux with mRFP-GFP-LC3 construct. The GFP signal was easily quenched in autolysosome as the acidic pH lysosomal background because of lysosomal hydrolysis, while mRFP fluorescence existed in the acidic pH background. The merged yellow signal GFP^+^ mRFP^+^ was visualized using a confocal microscope in the autophagosomes and GFP^-^ mRFP^+^ signal was visualized using a confocal microscope in the autolysosomes as described previously (Tang et al., 2013). (C) PLP2-TM activates autophagosome formation but blocks its fusion with lysosomes. HEK293T cells were co-transfected with mRFP-GFP-LC3 and PLP2-TM. As the positive control for induction of autophagy, HEK293T cells were transfected with mRFP-GFP-LC3 and then treated with complete medium supplemented with 400 nmol/L Rapamycin for 6 h. For the inhibition of autolysosome maturation, HEK293T cells were transfected with the plasmids of mRFP-GFP-LC3 and then treated with complete medium supplemented with 50 µmol/L CQ for 6 h. At 48 h post-transfection, the cells were fixed and assessed for GFP and mRFP fluorescence. Based on differential pH sensitivity of mRFP and GFP, the mRFP-GFP-LC3 probe differentiates between early, nonacidified autophagosomes (red^+^green^+^, yellow in merged images) from acidified, degradative autolysosomes (red^+^green^−^, red in merged images)

The terminal step in autophagy is the fusion of autophagosomes with lysosomes to form autolysosomes and subsequent degradation of the content. We wondered whether the PLP2-TM-induced accumulation of autophagosomes resulted in enhanced autophagic degradation. To answer this question, we measured the protein levels of a well-characterized autophagic substrate, p62/SQSTM1, which binds to LC3 and is specifically degraded as a result of complete autophagic flux (Bjorkoy et al., 2005; Pankiv et al., 2007), in PLP2-TM-expressing HEK293T cells by immunoblotting. Although the abundance of LC3-II was upregulated by PLP2-TM, which is indicative of increased autophagic activity (Fig. 5A, compare lane 2 vs. lane 1 in LC3-I/II panel), the level of the p62 was not reduced but rather slightly increased (compare lane 2 vs. lane 1 in top panel). In contrast, Rapamycin, which induced complete autophagy, not only increased LC3-II levels but also caused a significant decline in p62 expression (compare lane 3 vs. lane 1). Furthermore, the decreased p62 expression that induced by Rapamycin was recovered by PLP2-TM (compare lane 4 vs. lane 3, Fig. 5A). These data reveal that PLP2-TM induces incomplete autophagy, with a disconnection between autophagosome accumulation and autophagic cargo hydrolysis, implying that PLP2-TM may interfere with the fusion of autophagosomes with lysosomes or impair the autolysosomal degradation.

To further clarify the mechanism by which PLP2-TM induces incomplete autophagy, we took advantage of a tandem-tagged fluorescent reporter, mRFP-GFP-LC3 (Ke and Chen, 2011; Kimura et al., 2007; Klionsky et al., 2012; Mizushima et al., 2010) (Fig. 5B). This reporter construct encodes a fusion protein composed of three open reading frames (ORFs), i.e., mRFP, GFP and LC3 (Amino- to Carboxyl-terminal order). In transfected cells, when autophagosomes fuse with lysosomes, the GFP-green fluorescence of the mRFP-GFP-LC3 fusion protein is quenched in the acidic autolysosomal environment, while red fluorescence emitted from mRFP (which is relatively acid-resistant) remains. By contrast, when fusion of mRFP-GFP-LC3-autophagosomes with lysosomes is interrupted, the mRFP-GFP-LC3-autophagosomes emit both green GFP- and red mRFP-fluorescences which colocalize as yellow puncta in merged fluorescent images (Tang et al., 2013) (Fig. 5B). Validating this reporter system in our hands, we first showed that Rapamycin treated, mRFP-GFP-LC3-expressing cells which underwent complete autophagic flux indeed exhibited predominant red fluorescence with only dim green fluorescence (Fig. 5C). In contrast, cells co-expressing PLP2-TM and mRFP-GFP-LC3 displayed strong accumulation of mRFP-GFP-LC3-puncta which emitted colocalized red and green fluorescence (shown as yellow puncta in merged images, Fig. 5C). The fluorescence pattern of mRFP-GFP-LC3 in PLP2-TM-expressing cells closely resembled that of cells treated with cholorquine (Fig. 5C), a potent inhibitor of autophagosome-lysosome fusion. These findings indicate that PLP2-TM may impair the autophagosome maturation process by interfering with its fusion with lysosomes.

### PLP2-TM colocalizes and interacts with LC3

**Figure 6:**
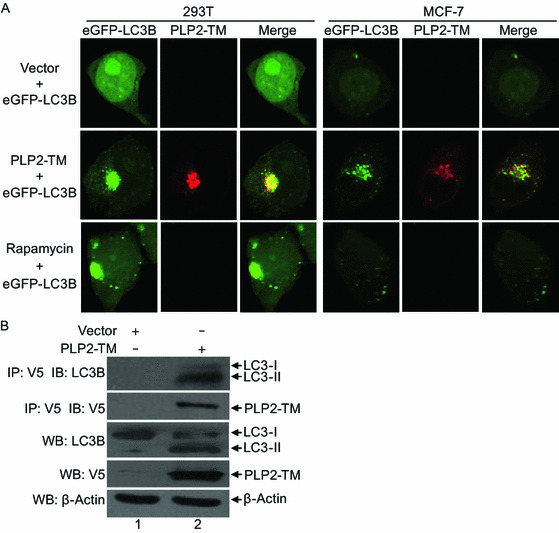
**PLP2-TM colocalizes and coimmunoprecipitates with autophagy marker protein LC3**. (A) HEK293T and MCF-7 cells were transfectd with pcDNA3.1 empty vector, PLP2-TM-V5. After 48 h post-transfection, the cells were fixed and incubated with anti-V5-tagged primary antibody, followed by being stained with an Alexa Fluor 594-conjugated goat anti-rabbit secondary antibody. Colocalization of PLP2-TM-V5 (red) and eGFP-LC3B (green) were observed using a confocal microscope as described in Fig. 1B. (B) HEK293T cells were transfected with pcDNA3.1 empty vector or PLP2-TM-V5 for 48 h, and the cell lysate was immunoprecipitated (IP) with an anti-V5 antibody and immunoblotted (IB) with an anti-LC3 and anti-V5-tagged antibody to detect expression of LC3 (top panel) and V5-tagged PLP2-TM (second panel), respectively. The whole cell lysate (WCL) was blotted with indicated antibodies to evaluate expression of endogenous LC3 and V5-tagged PLP2-TM. Beta-actin was analyzed to serve as a protein loading control

The lipidation of LC3 plays a crucial role in the formation of autophagosomes (Glick et al., 2010; Kabeya et al., 2000; Weidberg et al., 2011). In our efforts to further delineate the mechanisms underlying the induction of autophagy by PLP2-TM, we explored the possibility that PLP2-TM may physically associate with LC3, thereby regulating LC3-mediated phagophore elongation/closure. Confocal fluorescence microscopy revealed PLP2-TM substantially co-localized with eGFP-LC3 as cytoplasmic puncta in cotransfected HEK293T cells (Fig. 6A, left panels), indicating that PLP2-TM is in close proximity with LC3 on autophagosome membranes. This was not a cell-type specific phenomenon, as it was also observed in MCF-7 cells (Fig. 6A, right panels). Co-immunoprecipation analyses showed that V5-tagged PLP2-TM formed a complex with endogenously expressed LC3 (Fig. 6B). Interestingly, significantly more LC3-II than LC3-I was present in PLP2-TM immunoprecipitates, suggesting a higher affinity of LC3-II for PLP2-TM than that of LC3-I.

### PLP2-TM interacts with Beclin1 and promotes Beclin1-STING association

**Figure 7:**
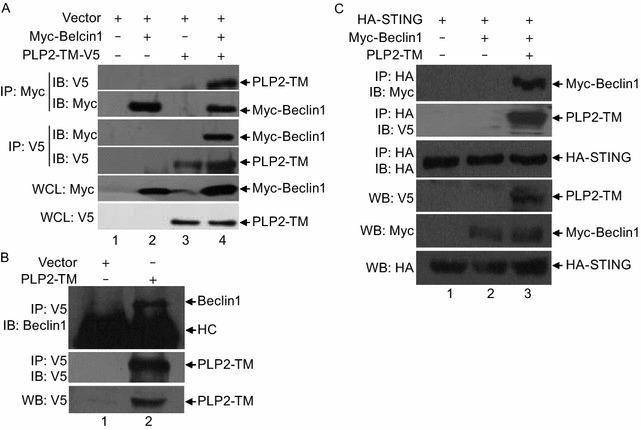
**PLP2-TM interacts with Beclin1 to promote Beclin1-STING interaction**. (A) PLP2-TM interacts with exogenous Beclin1. HEK293T cells were transfected with Myc-Beclin1 and pcDNA3.1 or PLP2-TM-V5 for 48 h. The cell lysate was subjected to immunoprecipitate (IP) with an anti-Myc antibody and immunoblot (IB) with an anti-V5 and anti-Myc antibodies (upper and second panels). The cell lysate was also subjected to immunoprecipitate (IP) with an anti-V5 antibody and immunoblot (IB) with an anti-Myc and anti-V5 antibodies (the third and fourth panels). The whole cell lysate (WCL) was blotted with indicated antibodies to evaluate expression of Myc-Beclin1 and V5-tagged PLP2-TM (the fifth and bottom panels). (B) PLP2-TM is associated with endogenous Beclin1. HEK293T cells were transfected with pcDNA3.1 or PLP2-TM-V5 for 48 h. The cell lysate was prepared to immunoprecipitate (IP) with an anti-V5 antibody and immunoblot (IB) with an anti-Beclin1 and an anti-V5 antibody (upper and second panels). The whole cell lysate (WCL) was blotted with indicated antibody to evaluate expression of V5-tagged product (bottom panel). HC indicates heavy chain of immunoglobulin. (C) PLP2-TM promotes the interaction between Beclin1 and STING. HEK293T cells were co-transfected with Myc-Beclin1, HA-STING and PLP2-TM-V5 for 48 h. The cell lysate was immunoprecipitated (IP) with an anti-HA antibody and immunoblotted (IB) with an anti-Myc (upper panel), anti-V5 (the second panel) and an anti-HA antibody (the third panel). The whole cell lysate (WCL) was analyzed to evaluate expression of each epitope-tagged product with the indicated antibodies (bottom panel)

As a critical regulator of the autophagic process, Beclin1 functions as a scaffold for assembly of the PI3K complexes catalyzing early autophagy. In addition, it orchestrates the late stages of autophagy through interacting with UVRAG, Rubicon to modulate the maturation of autophagosomes to autolysosomes (Jung et al., 2009; Matsunaga et al., 2009; Pattingre et al., 2008; Zhong et al., 2009a; Zhong et al., 2009b). Our data that PLP2-TM not only promoted autophagosome formation but also impacted on fusion of autophagosomes with lysosomes prompted us to determine whether Beclin1 is a target for PLP2-TM. In co-IP experiments, V5-tagged PLP2-TM was found to associate with Myc-tagged Beclin1 reciprocally in co-transfected HEK293T cells (Fig. 7A). Furthermore, PLP2-TM physically interacted with endogenous Beclin1 (Fig. 7B, lane 2 in top panel), suggesting that Beclin1 expressed at physiologic levels also forms a complex with PLP2-TM.

Previously, we have shown that PLP2-TM associated with STING, a key regulator of antiviral IFN signaling (Sun et al., 2012a). In light of the emerging roles for autophagy and its components in regulating innate immunity (Levine et al., 2011), we determined whether Beclin1 is involved in the regulation of STING by PLP2-TM. While HA-tagged STING did not co-immunoprecipiate with Myc-tagged Beclin1, these two proteins formed a complex when PLP2-TM was co-expressed (Fig. 7C). These data support the notion that the ability to interact with Beclin1 and regulate autophagy may be linked to the PLP2-TM control of innate immune signaling.

### Knockdown of Beclin1 partially reverses PLP2-TM’s inhibitory effect on innate immunity and is detrimental to PEDV replication

**Figure 8:**
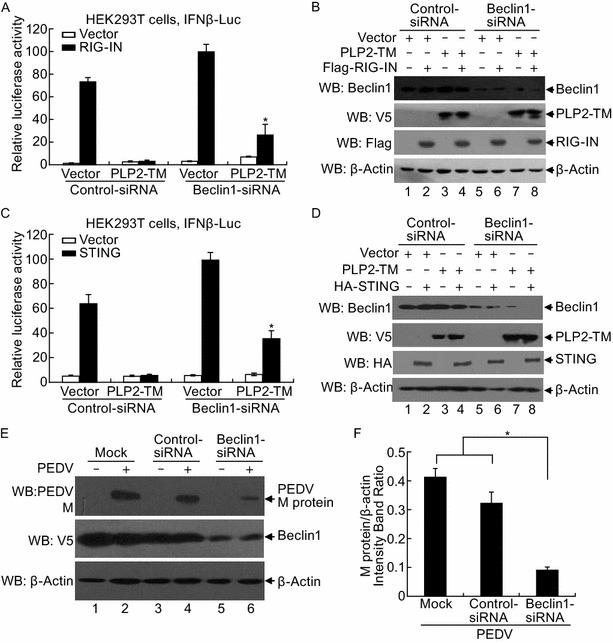
**Beclin1 is required for the PLP2-TM negative regulation of IFN expression and contributes to coronavirus replication**. (A, C) HEK 293T cells were mock transfected or transfected with control-siRNA, and Beclin1-siRNA at a concentration of 100 nmol/L for 24 h, and then the cells were transfected with IFNβ-luc reporter, pRL-TK, with expressing construct of RIG-IN (A) or STING (C) or empty vector and PLP2-TM for another 24 h. Cells were incubated for 48 h and firefly luciferase and Renilla luciferase activities were assayed. The results were expressed as mean relative luciferase (firefly luciferase activity divided by Renilla luciferase activity) with a standard deviation from repeated experiments carried out in triplicate. For statistical analysis, the data of Vector or PLP2-TM between Beclin1 siRNA and control siRNA were subjected to unpaired, two-tailed Student’s *t* test using the Microsoft SPSS 12.0 software, and a *P* value < 0.05 or less was considered statistically significant difference. (B and D) Proteins were extracted from the cells in Fig. 8A (B) or Fig. 8C (D) and analyzed using Western blotting with an anti-Beclin1 antibody to visualize Beclin1 proteins (top panel) and anti-V5 antibody (second panel) to visualize the PLP2-TM construct expression. Anti-Flag (B) and anti-HA (D) antibodies were used to visualize RIG-IN and STING proteins (third panel). Beta-actin was detected using Western blotting as protein loading control (bottom panel). (E) Beclin1-siRNA reduces PEDV replication in Vero cells. Vero cells were transfected with either Beclin1 siRNA or control siRNA at a concentration of 100 nmol/L for 24 h, and then the cells were treated by PEDV at a multiplicity of infection (MOI) of 0.1 for another 24 h. Cells were incubated for 48 h and the M protein expressions were assayed using Western blotting assay. (F) The optical density of M protein band in Fig. 8E was measured by densitometric analysis using ImageJ software and then the ratio of M protein/β-actin was calculated. For statistical analysis, the data between Beclin1 siRNA and control siRNA or mock control were subjected to unpaired, two-tailed Student’s *t* test using the Microsoft SPSS 12.0 software, and a *P* value < 0.05 or less was considered statistically significant difference

To understand the biological significance of the PLP2-TM regulation of autophagy, we studied the effects of Beclin1 knockdown on innate immune signaling and on coronavirus replication. In control siRNA transfected cells, activation of the IFN-β promoter by the constitutively active RIG-I CARD (RIG-IN) was ablated by PLP2-TM. This was in line with our previous publications (Clementz et al., 2010; Devaraj et al., 2007; Sun et al., 2012a). We found that knock-down of Beclin1 slightly and not significantly increased the IFNbeta-Luc reporter activity when cotransfected with vector. Remarkably, knockdown of Beclin1 partially, but significantly reversed PLP2-TM blocking RIG-IN-activated IFN expression (Fig. 8A). Immunoblotting demonstrated that Beclin1 siRNA efficiently depleted the target without affecting PLP2-TM expression (Fig. 8B). Similarly, activation of the IFN-β promoter by STING was inhibited by PLP2-TM in control siRNA transfected cells. However, knockdown of Beclin1 partially, but significantly reversed the inhibition that induced by PLP2-TM (Fig. 8C). The data was also showed that Beclin1 siRNA efficiently knocked down the target of Beclin1 without affecting PLP2-TM expression (Fig. 8D). Next, we tested how silencing of Beclin1 affected coronavirus replication. To achieve this goal, we took advantage of a Vero cell culture model for PEDV, the PLP2-TM of which also promotes autophagy (Fig. 3A). While transfection of a control siRNA had no impact on M protein expression in PEDV infected cells, siRNA-mediated knockdown of Beclin1 significantly diminished M protein production (Fig. 8E and 8F). Taken together, these data suggest that coronaviral PLP2-TM exploits Beclin1 and the autophagy pathway to attenuate innate antiviral responses and concomitantly facilitate viral replication.

## Discussion

Many viruses have evolved to exploit the autophagic machinery to their own benefit (Kudchodkar and Levine, 2009; Orvedahl and Levine, 2008; Shoji-Kawata and Levine, 2009), and coronaviruses are no exception. A number of studies have shown that autophagy is induced during infections by various coronaviruses, although controversial results have been reported concerning whether autophagy is required for coronavirus replication (de Haan and Reggiori, 2008; Prentice et al., 2004; Reggiori et al., 2010; Zhao et al., 2007). At present, the underlying mechanisms by which coronaviruses promote autophagy are poorly understood. The nsp6 encoded by infectious bronchitis virus, an avian coronavirus, was recently reported to induce autophagosome formation, as were the nsp6 homologues encoded by MHV, SARS-CoV and the closely related arterivirus PRRSV (Cottam et al., 2011). We present evidence in this study that expression of the membrane-anchored coronavirus papain-like protease PLP2 domain (and its homologues) alone is capable of activating autophagy in nutrient-rich conditions, assigning a novel function to this multifunctional viral protein which is known to act as a viral protease, a DUB enzyme, and an IFN antagonist (Barretto et al., 2005; Chen et al., 2007; Clementz et al., 2010; Devaraj et al., 2007; Sun et al., 2012a). Importantly, we have demonstrated this in multiple cell types (HEK293T, HeLa and MCF-7), and shown it to be an attribute shared by PLP2-TM/PLpro-TM of different coronaviruses, including HCoV-NL63, SARS-CoV, MERS-CoV and PEDV. This finding uncovers a previously unappreciated role for PLP2-TM/PLpro-TM in regulation of autophagy by coronaviruses and may provide novel insights into the mechanisms of coronavirus pathogenesis.

Our data show that the PLP2 domain and the downstream hydrophobic TM motif are both needed to promote autophagy. Neither PLP2 nor TM alone is sufficient, as evidenced by the inabilities of soluble PLP2 and PLP1-TM to induce autophagosome (LC3 puncta) formation (Data not shown). Mechanistically, we found that PLP2-TM physically interacted with Beclin1 and LC3, both of which are involved in the early steps of autophagosome formation (Kraft and Martens, 2012; Mehrpour et al., 2010). Interestingly, our data also reveal that PLP2-TM induces incomplete autophagy that does not culminate in autophagosome maturation to autolysosomes. Evidence supporting this notion came from the experiments showing that degradation of the autophagic substrate p62/SQSTM1 was retarded and that the autolysosome-liable GFP fluorescence of the mRFP-GFP-LC3 reporter protein was not lost in spite of enhanced LC3 lipidation. Beclin1, again, is likely the target responsible for the deficient autophagosome maturation in PLP2-TM expressing cells, given its involvement in the UVRAG-containing PI3K complex that controls fusion between autophagosmes and lysosomes (Kang et al., 2011; Liang et al., 2008). Of note, accumulating evidence suggests that Beclin1 is a prime target for viruses that manipulate the autophagy pathway (Munz, 2011). For example, Influenza A virus M2 and HIV Nef bind to Beclin1 to hamper the fusion of autophagosomes with lysosomes (Gannage et al., 2009; Kyei et al., 2009). We propose that the coronavirus PLP2-TM adopts a similar strategy to impede the maturation of autophagic vacuoles. However, the precise mechanism will need to be further studied. Regardless, the induction of incomplete as opposed to complete autophagy by PLP2-TM may represent an evolutionary advantage of the virus, in that it prevents autophagic degradation of viral products generated in infected cells, promoting maximal viral survival.

The findings that nsp6 (Cottam et al., 2011) and PLP2-TM/PLpro-TM of coronaviruses promote autophagy argue that the autophagy pathway and/or autophagy-related protein(s) actively participate in coronavirus-host interactions. A tempting hypothesis is that autophagy may facilitate viral propagation, as demonstrated for other positive-strand RNA viruses such as dengue virus, and Poliovirus (Richards and Jackson, 2013). However, experimental evidence concerning whether autophagy is required for replication of coronaviruses has been contradictory. While earlier studies suggested autophagy to play a role (Cottam et al., 2011; Prentice et al., 2004), several latest reports argued against it (Maier and Britton, 2012; Maier et al., 2013; Reggiori et al., 2010). These recent studies support a model in which MHV and arterivirus EAV hijack the LC3-I-positive EDEMsomes, rather than autophagosome membranes, to form the DMVs in which viral replication takes place (Monastyrska et al., 2013; Reggiori et al., 2010). However, the possibility that different coronaviruses may have a disparate requirement for autophagy for optimal viral replication cannot be ruled out. Supporting this notion, several studies have shown that autophagy is induced by infection with a closely related arterivirus, PRRSV, whose replication is crippled by inhibition of autophagy (Chen et al., 2012; Liu et al., 2012; Sun et al., 2012b). We show here that knockdown of the master regulator of autophagy, Beclin1, impairs PEDV M protein production. Clearly, more studies with a greater number of different coronaviruses are warranted (Maier and Britton, 2012).

Another intriguing finding of this study is that autophagy is subverted by coronaviral PLP for immune evasion. We have previously shown that coronavirus PLpro-TM/PLP2-TM negatively regulates antiviral defenses by inhibiting the activation of IRF3 (Clementz et al., 2010; Devaraj et al., 2007), and that the PLPs disrupt STING-mediated IFN induction (Sun et al., 2012a), but the mechanism(s) by which coronavirus PLPs target STING (and possibly other signaling molecules) to inhibit IRF3-dependent antiviral response is unknown. In this work, we have demonstrated that PLP2-TM promotes the interaction of STING with Beclin1, but not with LC3 (data not shown), suggesting that PLP2-TM may sequester STING to autophagosomes through Beclin1, thereby inhibiting downstream innate immune signaling. Although not tested in this study, PLP2-TM-induced autophagy may also segregate other innate immune signaling components and perhaps even viral dsRNAs from ligation to cytoplasmic viral sensors. Supporting the exploit of Beclin1 and autophagy for viral immune escape, we found that knockdown of Beclin1 (and thus autophagosome formation) partially but significantly reversed the blockade of PLP2-TM on activation of the IFN response. The reason the relief was only partial was likely due to incomplete depletion of Beclin1 and existence of autophagy-independent immune evasion mechanisms, as exemplified by the deubiquitination of RIG-I, TRAF3, etc. by PLPs (Sun et al., 2012a).

In summary, results of this study demonstrate that the coronavirus papain-like proteases along with their transmembrane anchors activate incomplete autophagy by interacting with Beclin1. Autophagosomes induced by CoV PLP2-TM/PLpro-TMs may provide a platform for viral targeting Beclin1 to sequester STING and possibly other critical innate signaling components to impede downstream antiviral responses, thereby promoting viral replication. Further studies will be needed to elucidate the precise mechanisms of autophagosome induction by CoV PLPs and the exact roles that autophagy plays in coronavirus replication, antiviral innate immune responses and disease pathogenesis.

## Materials and methods

### Cells, plasmids, siRNAs, antibodies and other reagents

HEK293T, HeLa and MCF-7 cells were cultured in Dulbecco’s modified Eagle’s medium (DMEM) (Gibco, Cat. No.12800-017) supplemented with 10% (*v*/*v*) fetal calf serum (Gibco, Cat. No. 1036489), 100 U/mL of penicillin and 100 µg/mL of streptomycin. DNA constructs encoding HCoV-NL63 PLP2-TM, SARS-CoV PLpro-TM, PEDV PLP2-TM, MERS-CoV PLpro-TM and plasmid of HA-STING have been described (Clementz et al., 2010; Devaraj et al., 2007; Sun et al., 2012a; Yang et al., 2014). To construct a Myc-tagged Beclin1 expression vector, cDNA encoding the full-length (1353-bp long) human Beclin1 (GenBank ID: KJ395945-KJ395992) was amplified by reverse transcription-PCR (RT-PCR) from human mesenchymal stem cells (MSCs) and cloned into the pCMV-Myc vector (Clontech). The following PCR primers were used: 5′-CCCGAATTCGGATGGAAGGGTCTAAGACGTCC-3′ (forward primer) and, 5′-CGCGGTACCTCATTTGTTATAAAATTGTGAGGA-3′ (reverse primer). The plasmid encoding eGFP-LC3B was kindly provided by Dr. Songshu Meng (Yangzhou University, Jiangsu, China) (Kimura et al., 2007; Mizushima et al., 2010). A vector encoding tandem mRFP-GFP-LC3 was kindly provided by Dr. Shaobo Xiao (Huazhong Agricultural University, Wuhan, China) (Kimura et al., 2007; Mizushima et al., 2010). Beclin1 siRNA sequence and control siRNA sequence were described as previously (Hoyer-Hansen et al., 2005). The following primary antibodies were used for various protein analyses: anti-LC3 (Sigma-Aldrich, Cat. No. L7543); anti-p62 (MBL, Cat. No. PM045), anti-Beclin1(MBL, Cat. No. PD017), anti-V5 (MBL, Cat. No. PM003), anti-HA (MBL, Cat. No. 561) and anti-Myc (MBL, Cat. No. M047-3); anti-actin (Beyotime, Cat. No. AA128); chicken anti-V5 (Abcam, Cat. No. ab9113). Donkey anti-chicken Cy3 (Millipore, Cat. No. AP194C) and Alexa Fluor 594-conjugated goat anti-rabbit secondary antibodies (ZSGB-BIO, Cat. No. ZF-0516) were obtained from Millipore and ZSGB-BIO, respectively. Rapamycin (Sigma-Aldrich, Cat. No. R8781), Chloroquine (CQ) (Sigma-Aldrich, Cat. No. C6628) and 3-Methyladenine (3-MA) (Sigma-Aldrich, Cat. No. M9281) were obtained from Sigma-Aldrich.

### Confocal microscopy

HEK293T, HeLa or MCF-7 cells were grown on glass coverslips in 6-well plates. For the detection of autophagosomes, plasmid DNA expressing eGFP-LC3B or mRFP-GFP-LC3 (1 μg per well) was transfected in the presence or absence of 1 μg of various PLP-encoding vectors using Lipofectamine 2000 (Invitrogen, Cat. No. 11668-027) according to the manufacturer’s protocol. As a positive control to visualize the induction of autophagy, HEK293T cells were transfected with eGFP-LC3B for 48 h and then treated with 400 nmol/L of Rapamycin in complete culture medium for 6 h. The fluorescence of GFP-LC3 was observed under a Zeiss LSM-510 confocal fluorescence microscope. Cells containing three or more GFP-LC3 dots were defined as autophagy-positive cells. The percentage of cells with characteristic punctate GFP-LC3 fluorescence relative to all GFP-positive cells was calculated as described previously (Li et al., 2011; Wong et al., 2008). Three random fields, each containing at least 80 GFP-positive cells, were counted, and three independent experiments were performed.

### Immunofluorescence

To evaluate the subcellular localization of eGFP-LC3B, mRFP-GFP-LC3 and NL63 PLPs, plasmid DNA expressing eGFP-LC3B or mRFP-GFP-LC3 (1 μg per well) was transfected in the presence or absence of 1 μg of various PLP-encoding vectors using Lipofectamine 2000 (Invitrogen, Cat. No. 11668-027) according to the manufacturer’s protocol. As a positive control to visualize the induction of autophagy, HEK293T cells were transfected with eGFP-LC3B for 48 h and then treated with 400 nmol/L of Rapamycin in complete culture medium for 6 h. In some experiments, HEK293T cells co-transfected with the plasmids of mRFP-GFP-LC3 and PLP2-TM for 48 h were treated with 50 μmol/L of CQ in complete medium for 6 h to inhibit autolysosome maturation. At indicated time points post transfection, cells were fixed with 4% formaldehyde in PBS for 10 min at room temperature. Cells were then incubated with 1:200 dilution of rabbit anti-V5 (MBL, Cat. No. PM003) or chicken anti-V5 (abcam, Cat. No. ab9113) in ADPS (PBS + 0.1% Triton-X100 + 5% fetal calf serum) for 1 h at room temperature. Following three PBS washes, cells were incubated with 1:200 dilution of Alexa Fluor 594-conjugated goat anti-rabbit (ZSGB-BIO, Cat. No. ZF0136) or donkey anti-chicken Cy3 (Millipore, Cat. No. AP194C) secondary antibody in ADPS for 1 h in dark. Following the incubation, cells were washed three times with PBS, mounted, and examined under a Zeiss LSM-510 confocal microscope.

### Western blotting analysis

HEK293T, HeLa and MCF-7 cells were seeded into 24-well plates and incubated at 37°C for 18 h. Cells were subsequently transfected with PLP2-TM construct or empty vector using Lipofectamine 2000 reagent according to manufacturer’s instructions. At 48 h post-transfection, cells were lysed in a buffer containing 0.5% Triton X-100, 150 mmol/L NaCl, 12.5 mmol/L β-glycerolphosphate, 1.5 mmol/L MgCl_2_, 2 mmol/L EDTA, 10 mmol/L NaF, 1 mmol/L Na_3_VO_4_, 2 mmol/L DTT and protease inhibitor cocktail (Sigma,Cat. No. P8340). Cell extracts were clarified by centrifugation at 5000 ×*g* at 4°C for 10 min, and protein concentration of lysate determined using BCA Protein Assay kit (Bio-med, Cat. No. pp0101). Protein samples were mixed with 30 µL of 2× SDS-PAGE sample buffer, boiled for 10 min, separated on SDS-PAGE gel, and transferred onto a PVDF membrane. Blots were incubated with indicated primary antibodies, washed three times in 1× TBS-T buffer, and subsequently incubated with HRP-conjugated secondary antibodies (ZSGB-BIO, Cat. No. ZF0136, Cat. No. ZF0312). Antibody-antigen reactions were detected using Western Lighting Plus-ECL chemiluminescence reagents (Applygen, Cat. No. P1010).

### Co-immunoprecipitation (Co-IP) analysis

HEK293T cells were seeded in 100-mm dishes at a density of 1 × 10^6^ cells/dish. Twelve hours later, cells were transiently transfected with a total of 10 µg of empty vector or indicated expression plasmids using Lipofectamine 2000 (Invitrogen, Cat. No. 11668-027). At 48 h post transfection, whole cell lysates were prepared and their protein concentrations determined using the procedures described above (for Western blotting analysis). The protein concentrations in cell lysates were adjusted to 1 µg/µL, and 500 µL of each lysate was used for co-IP. Lysates were pre-cleared by adding 20 µL of protein A + G Agarose (Beyotime, Cat. No. P2021) and 1 µg of normal IgG and incubating for 2 h at 4°C, followed by spinning down the agarose beads. The pre-cleared supernatant was then incubated with the indicated primary antibody [anti-V5 (MBL, Cat. No. PM003) or anti-HA (MBL, Cat. No. 561)/anti-Myc (MBL, Cat. No. M047-3)] with rocking overnight at 4°C. Thereafter, the beads-antibody-antigen complex was pelleted and washed 3 times with 1 mL of lysis buffer. The protein complexes were then eluted from the beads in 30 µL of 2× SDS-PAGE sample buffer by boiling for 10 min. Samples were separated on SDS-PAGE and transferred to PVDF membranes for Western blotting.

### IFN-β reporter assay

12–18 h prior to transfection, HEK 293T were seeded in 24 well plates. At a confluence of 80%, the cells were transfected with the Beclin1 siRNA or control siRNA at the concentration of 100 nmol/L using JetPRIME (PolyPlus, Cat. No. 114-15). After 24 h, the cells were transfected using JetPRIME with 200 ng of IFNβ-Luc reporter plasmid encoding firefly luciferase and 20 ng of pRL-TK plasmid encoding Renilla luciferase for normalization along with 300 ng of empty DNA vector or RIG-I/STING-expressing construct and 300 ng of vector or PLP2-TM constructs. 24 h after DNA transfection, the cell extracts were prepared and Luciferase activity and Renilla luciferase activity were assayed using the Dual Luciferase Reporter System (Promega, Cat. No. E1910) in a Luminometer according to the supplier’s instructions. Data were shown as mean relative luciferase (firefly luciferase activity divided by Renilla luciferase activity) with standard deviation from repeated experiments that were carried out in triplicate. For statistical analysis, the data between Vector and PLP2-TM were subjected to unpaired, two-tailed Student’s *t* test using Microsoft SPSS 12.0 software, and *P*-values of <0.05 were considered to indicate statistical significance.

### Transmission electron microscopy (TEM)

HEK293T cells were seeded in 100-mm dishes at a density of 1 × 10^6^ cells/dish. Twelve hours later, cells were transiently transfected with a total of 10 µg of empty vector or the indicated expression plasmid using Lipofectamine 2000 (Invitrogen, Cat. No. 11668-027). As a positive control for induction of autophagy, HEK293T cells were treated with 400 nmol/L of Rapamycin in complete medium for 6 h. Cells were washed three times with PBS, trypsinized, and collected by centrifugation at 1000 ×*g* for 10 min. The cell pellets were fixed with 3% glutaraldehyde in 0.075 mol/L phosphate buffer (pH 7.4) for 2 h at 4°C. The cells were washed in the solution containing 0.075 mol/L phosphate and 0.19 mol/L sucrose three times for 10 min each and post-fixed in 1% OsO_4_ in 0.24 mol/L phosphate buffer (pH 7.4) for 2 h. After being washed for 15 min in 0.075 mol/L phosphate buffer and 0.19 mol/L sucrose buffer at 4°C, the cells were dehydrated with a graded series of ethanol and gradually infiltrated with epoxy resin. Samples were sequentially polymerized at 35°C for 12 h, 45°C for 12 h, and 60°C for 24 h. Ultrathin sections (about 70 nm) were cut using an LEICA microtome and mounted on copper slot grids. Sections were doubly stained with uranyl acetate for 10 min and lead citrate for another 10 min and observed under a Hitachi H-7650 transmission electron microscope.
